# Recurrent hypocalcaemic torsades de pointes due to hungry bone syndrome: a rare complication of thyroidectomy

**DOI:** 10.1007/s12471-020-01533-8

**Published:** 2021-01-04

**Authors:** Q. A. J. Hagdorn, P. Loh, S. Velthuis

**Affiliations:** 1 Department of Cardiology, Meander Medical Center, Amersfoort, The Netherlands; 2 Department of Cardiology, University Medical Center Utrecht, Utrecht University, Utrecht, The Netherlands

**Fig. 1:**
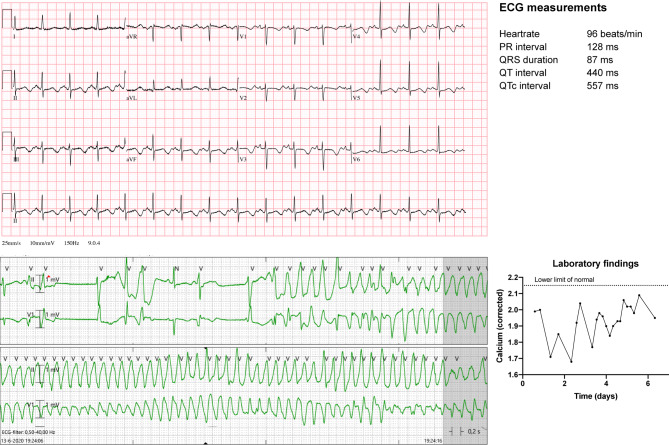
*Upper* Electrocardiogram demonstrating QT prolongation and ST‑T changes attributable to hypocalcaemia. *Lower left* Rhythm recording of the development of torsades de pointes ventricular tachycardia. *Lower right* Graph depicting the serum calcium levels over time

A 49-year-old, otherwise healthy female was admitted to our hospital for thyroidectomy. Postoperatively, presumably due to perioperative hypoperfusion of the parathyroid glands, she developed ‘hungry bone syndrome’, which is characterised by suppressed parathyroid hormone levels, resulting in an increased net bone uptake of calcium and subsequent severe hypocalcaemia [1]. Even with intravenous calcium supplementation, she developed QT prolongation and multiple episodes of torsades de pointes (TdP) ventricular tachycardia without cardiac output (Fig. 1). Isoprenaline was started to increase her heart rate to prevent further TdP. However, despite adequate heart rates (70–100 beats/min) and increasing, yet still subnormal, calcium levels, sustained TdP repeatedly developed, requiring electrocardioversion. She was transferred to a tertiary clinic, where her rhythm was successfully controlled by temporary tachypacing and high-dose beta-blockers. Chronic calcium supplementation eventually normalised her calcium levels and QT time.

Even though hypocalcaemia-induced TdP rarely complicates thyroidectomy [2–4], this case demonstrates the value of electrocardiographic and electrolyte screening after thyroidectomy and rhythm surveillance in severe hypocalcaemia.
